# Supplementary material for: Structural basis of adenylyl cyclase 9 activation
Source: Nat Commun. 2022 Feb 24;13:1045. doi: 10.1038/s41467-022-28685-y (PMC8873477; doi:10.1038/s41467-022-28685-y)
Supplement: Supplementary file 1 — Supplementary Information [file 41467_2022_28685_MOESM1_ESM.pdf]

Supplementary information

Structural basis of adenylyl cyclase 9 activation

Authors: Chao Qi[1,2], Pia Lavriha[2], Ved Mehta[1,2], Basavraj Khanppnavar[1,2], Inayathulla Mohammed[3], Yong Li[4], Michalis Lazaratos[5], Jonas V. Schaefer[6], Birgit Dreier[6], Andreas Plückthun[6], Ana-Nicoleta Bondar[5], Carmen W. Dessauer[4], Volodymyr M. Korkhov[1,2]*

Affiliations:

[1] Institute of Molecular Biology and Biophysics, ETH Zurich, Switzerland

[2] Laboratory of Biomolecular Research, Division of Biology and Chemistry, Paul Scherrer Institute, Villigen, Switzerland

[3] Biozentrum, University of Basel, Switzerland

[4] Department of Integrative Biology and Pharmacology, McGovern Medical School, University of Texas Health Science Center, Houston, TX, USA

[5] Freie Universität Berlin, Germany, Department of Physics, Theoretical Molecular Biophysics Group

[6] Department of Biochemistry, University of Zurich, Switzerland

* Correspondence to: volodymyr.korkhov@psi.ch.

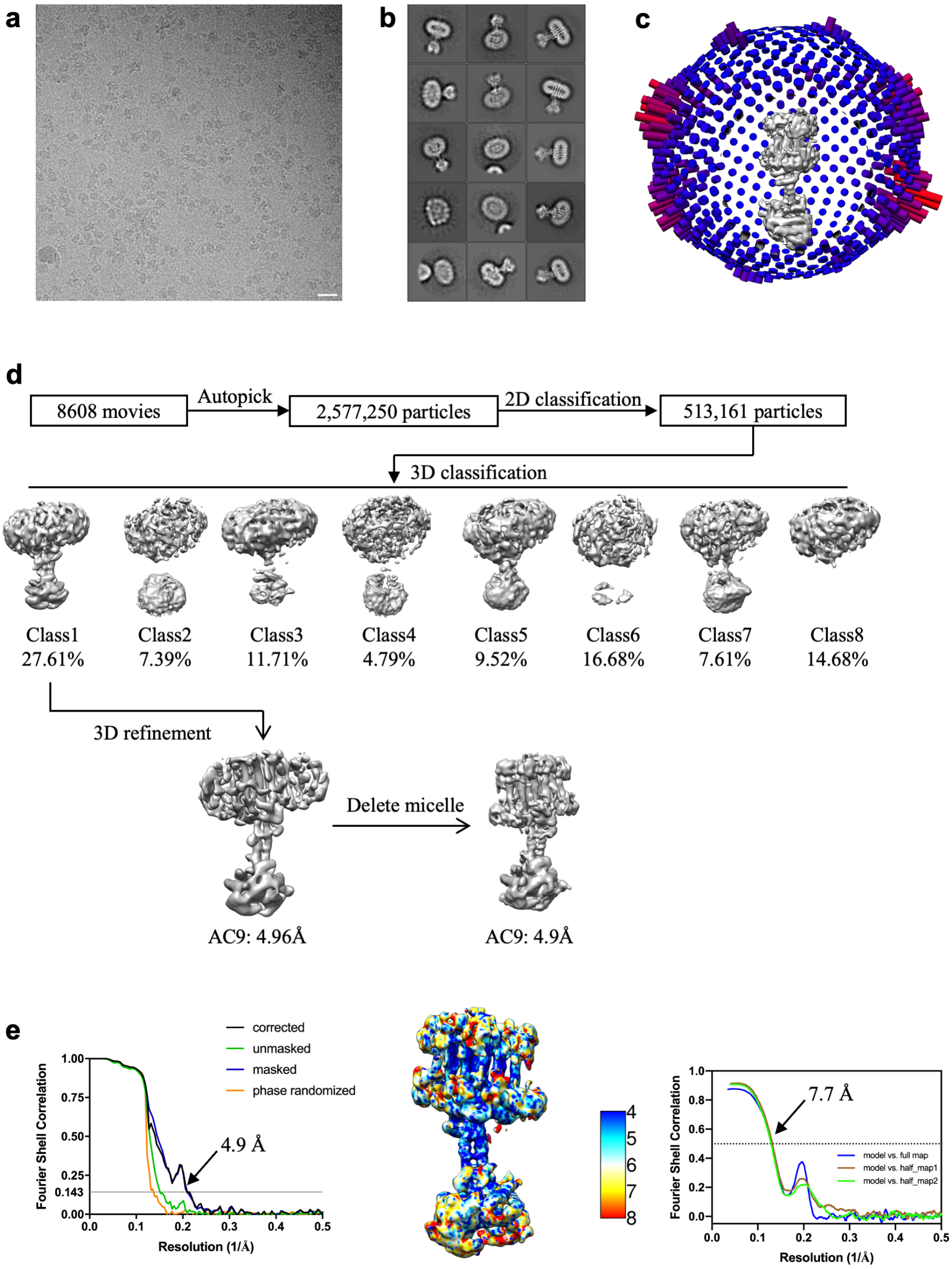

Supplementary Figure 1 | Cryo-EM image processing procedure of AC9-M dataset. a, A representative micrograph from 8608 micrograghs of AC9 in presence of 0.5 mM MANT-GTP; scale bar corresponds to 20 nm. **b**, 2D classes of AC9-M dataset show distinguishable features, including the detergent micelle and the protruding soluble domain. **c**, Angular distribution of AC9-M dataset. **d**, Overview of the AC9-M dataset image processing procedure, including particle picking, 2D and 3D classification and 3D auto-refinement. **e**, Fourier shell correlation plot (FSC) plots of the map AC9-M (left), local resolution estimation (middle) and map to model FSC plot (right).

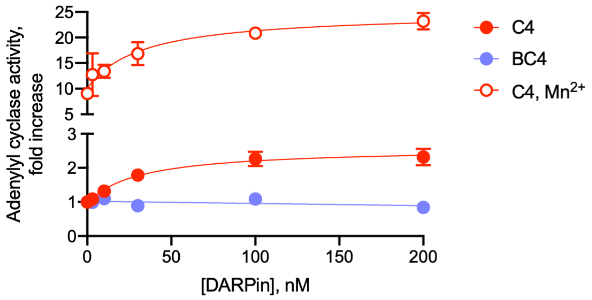

Supplementary Figure 2 | Adenylyl cyclase activity assays using AC9 in membrane preparations from Sf9 cells. The DARPin C4 or the negative control, DARPin BC4 (a DARPin selected as a binder for *Mycobacterium intracellulare* Cya/Rv1625c), were titrated into the reaction mixture to determine their effect on AC9 activity. Similar results were obtained in the presence of Mg^{2+} (closed circles) and Mn^{2+} (open circles) as counterions; n = 3 independent experiments (for BC4 n = 2); the data points are represented as mean ± S.E.M.

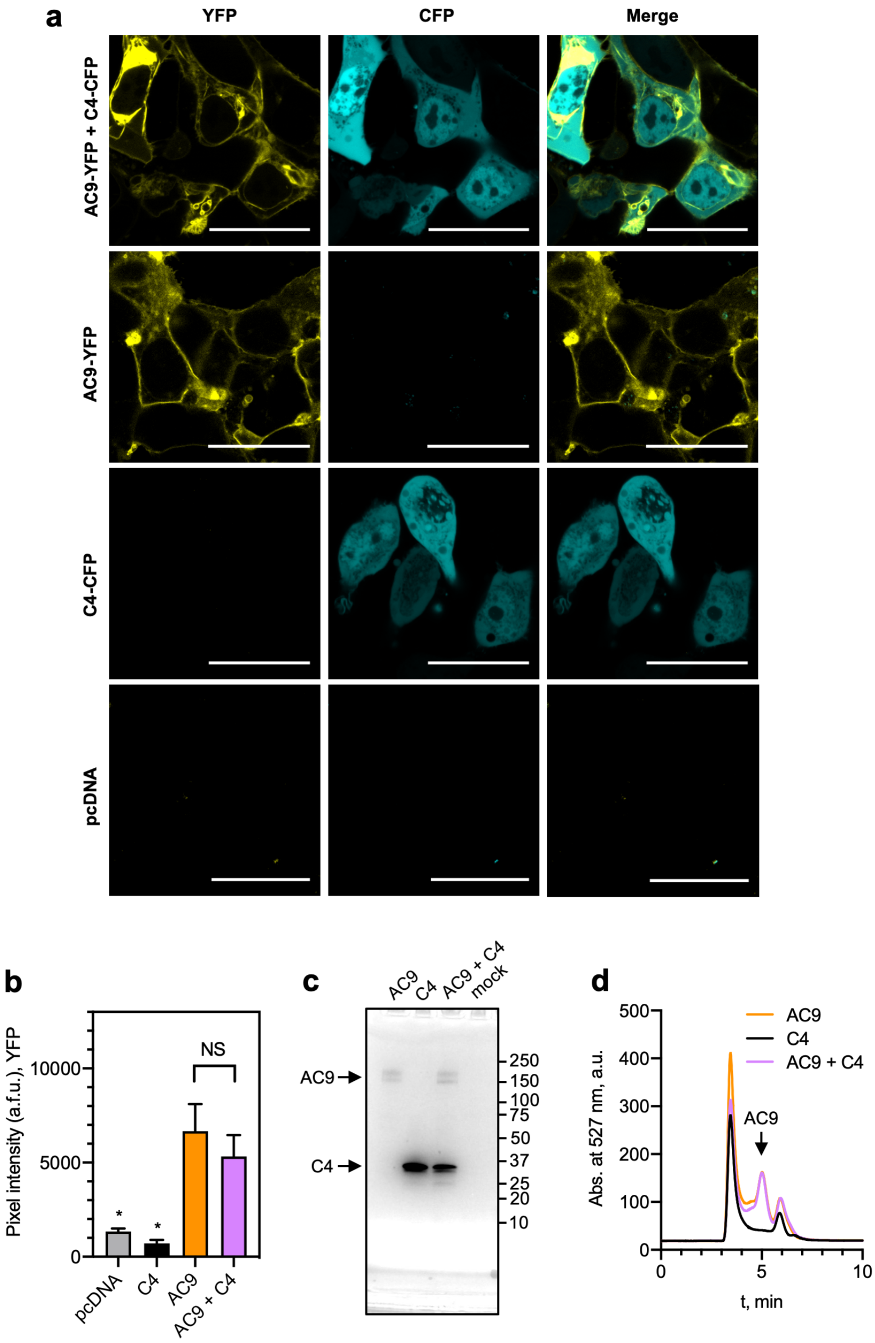

Supplementary Figure 3 | Effect of DARPin C4 on AC9 expression. a, Confocal imaging of HEK293F cells expressing AC9-YFP and/or DARPin C4-CFP constructs. The controls include the cells transfected with DARPin C4 alone or with pCDNA3.1 vector. The scale bar corresponds to 30 µm. **b,** Quantification of the imaging data shown in (a), based on pixel intensities measured at the plasma membrane (n = 7 individual cells). Statistical significance was assessed using ordinary one-way analysis of variance (ANOVA) (P = 0.00019, ***), followed by Tukey's multiple comparisons test, with P values of AC9 vs. AC9 + DARPin C4 of 0.7378 (NS), AC9 vs. DARPin C4/pcDNA of 0.0007/0.0024 (***/**), AC9 + DARPin C4 vs DARPin C4/pcDNA of 0.0091/0.0276 (**/*) and DARPin C4 vs. pcDNA of 0.9632 (NS); "NS" stands for "not significantly different"; "a.f.u." - arbitrary fluorescence units. The data are represented as mean ± S.E.M. **c,** SDS PAGE and in-gel fluorescence shows that AC9-YFP expressing is unaffected by the presence of DARPin C4-CFP construct; the numbers (in kDa) correspond to the molecular weight marker bands on the right side of the gel. **d,** Fluorescence size exclusion chromatography shows that the peak corresponding to solubilized AC9-YFP is not affected by the presence of a co-expressed DAPRin C4 (indicated by an arrow); "a.u." – absorbance units.

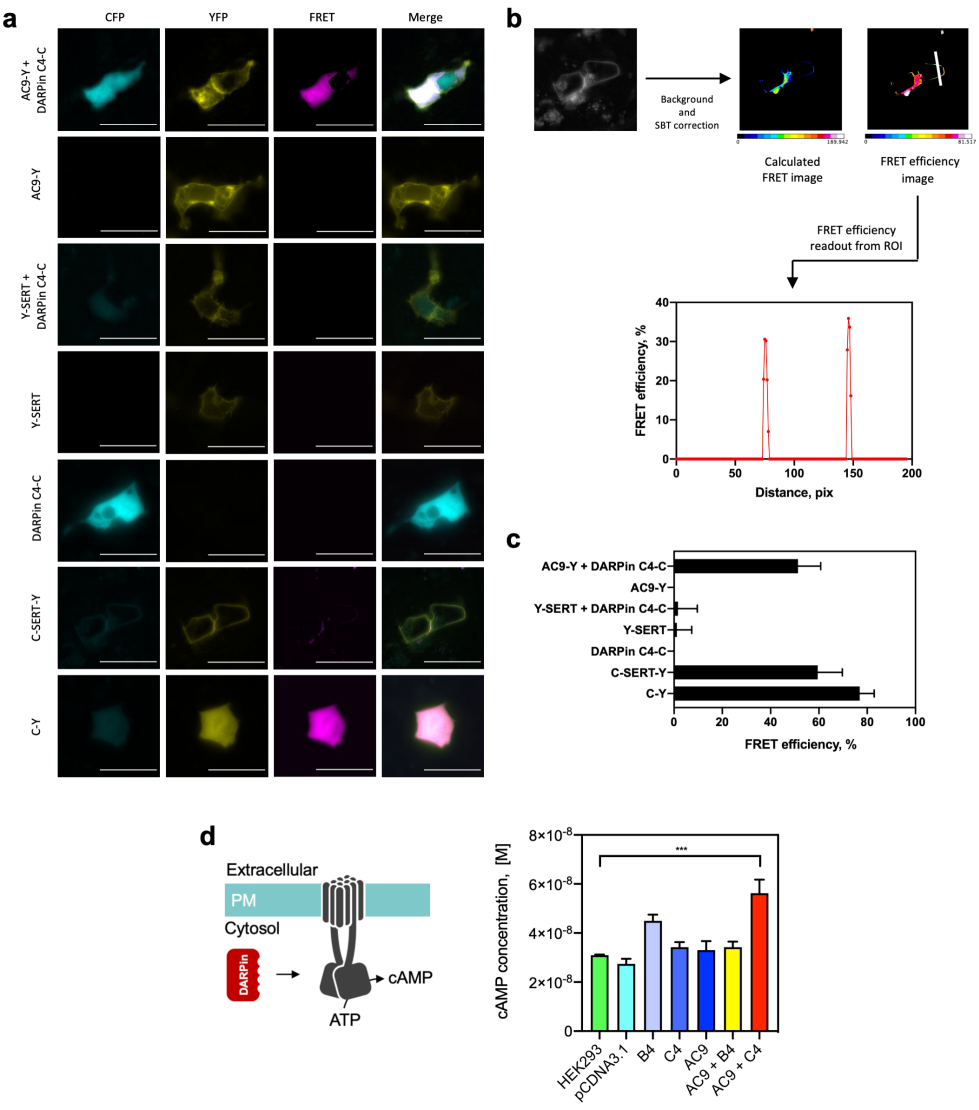

Supplementary Figure 4 | DARPin C4 interacts with AC9 in vivo. a, FRET microscopy reveals interactions between AC9-YFP and DARPin C4-CFP in HEK293F cells transfected with the corresponding plasmid DNA constructs. Individual constructs of AC9 and DARPin-C4, along with an unrelated protein, SERT (fluorescently labelled with YFP at the N-terminus), were used as negative controls. CFP-YFP (C-Y) and CFP-SERT-YFP (C-SERT-Y) were used as positive controls. The representative images are shown, corresponding to c. **b-c**, The image processing was performed as described under "Methods"; after background and spectral bleed-through (SBT) correction of the images, the calculated FRET images were used to calculate the FRET efficiency images, regions of interest were defined (corresponding to the plasma membrane for all constructs, and to the cytosolic regions for DARPin-C4-C and C-Y). The maximal FRET efficiency values were averaged and plotted as a bar graph (c). The values in c indicate mean ± S.E.M. (n = 30 cells). **d**, The sketch (*left*) indicates the activity assays performed using the AC9 and DAPRin-C4 constructs expressed in the HEK293F cells (YFP- and CFP-tagged versions of the proteins, respectively); n = 3 independent experiments (for B4 n = 2). (*Right).* The in vivo cAMP accumulation assays show that only in the presence of overexpressed AC9 and DAPRin C4 together there is significant production of cAMP in the HEK293F cells. The values were compared using one-way analysis of variance (ANOVA), followed by Dunnett's test indicates that out of all tested variations. Only the AC9 + C4 sample is significantly different from the control, P = 0.0003 (***); details are provided in the Source Data file.

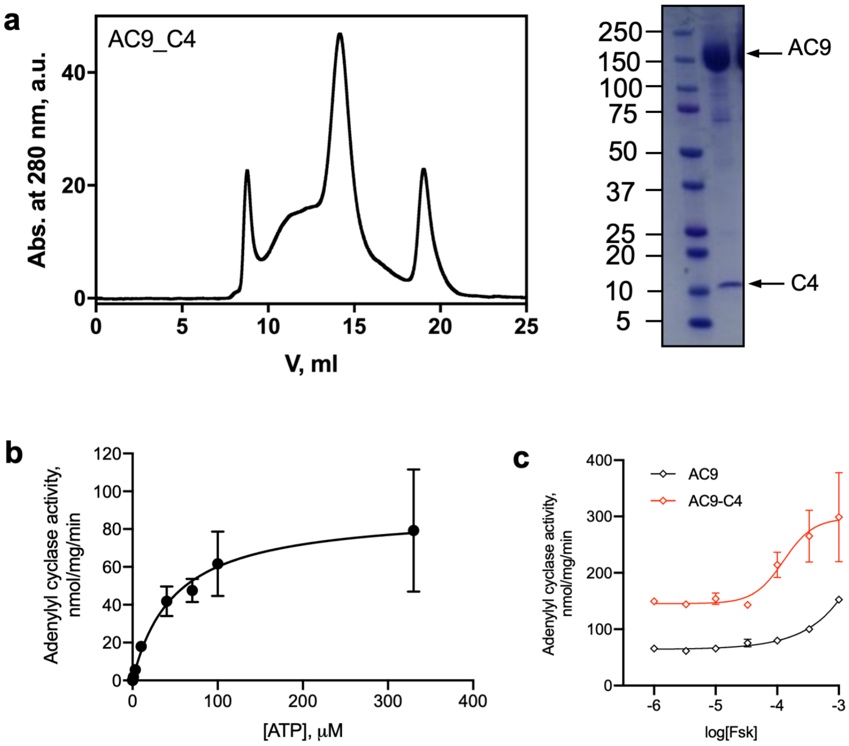

Supplementary Figure 5 | Purification and enzymatic activity of the AC9-C4 complex. a, Size-exclusion chromatography (SEC) of the AC9-C4 complex purified in digitonin (*left*) and SDS-PAGE of the purified AC9-C4 complex (*right*). The proteins in the complex are indicated with arrows; the numbers (in kDa) correspond to the molecular weight marker bands on the left side of the gel; "a.u." – absorbance units. **b,** Enzyme kinetics of the AC9-C4 complex reveals a K_m of 49 µM and a V_{max} 90 nmol/mg/min (n = 3 independent experiments). **c,** Adenylyl cyclase activity of AC9 or AC9-C4 complex in the presence of increasing concentrations of forskolin (Fsk) show behavior similar to that observed for AC9-Gαs complex (*1*). Forskolin does not efficiently activate AC9 alone, but activates the AC9-DARPin C4 complex with an EC_{50} of ~130 µM (n = 3 independent experiments). The data in b and c are shown as mean ± S.E.M.

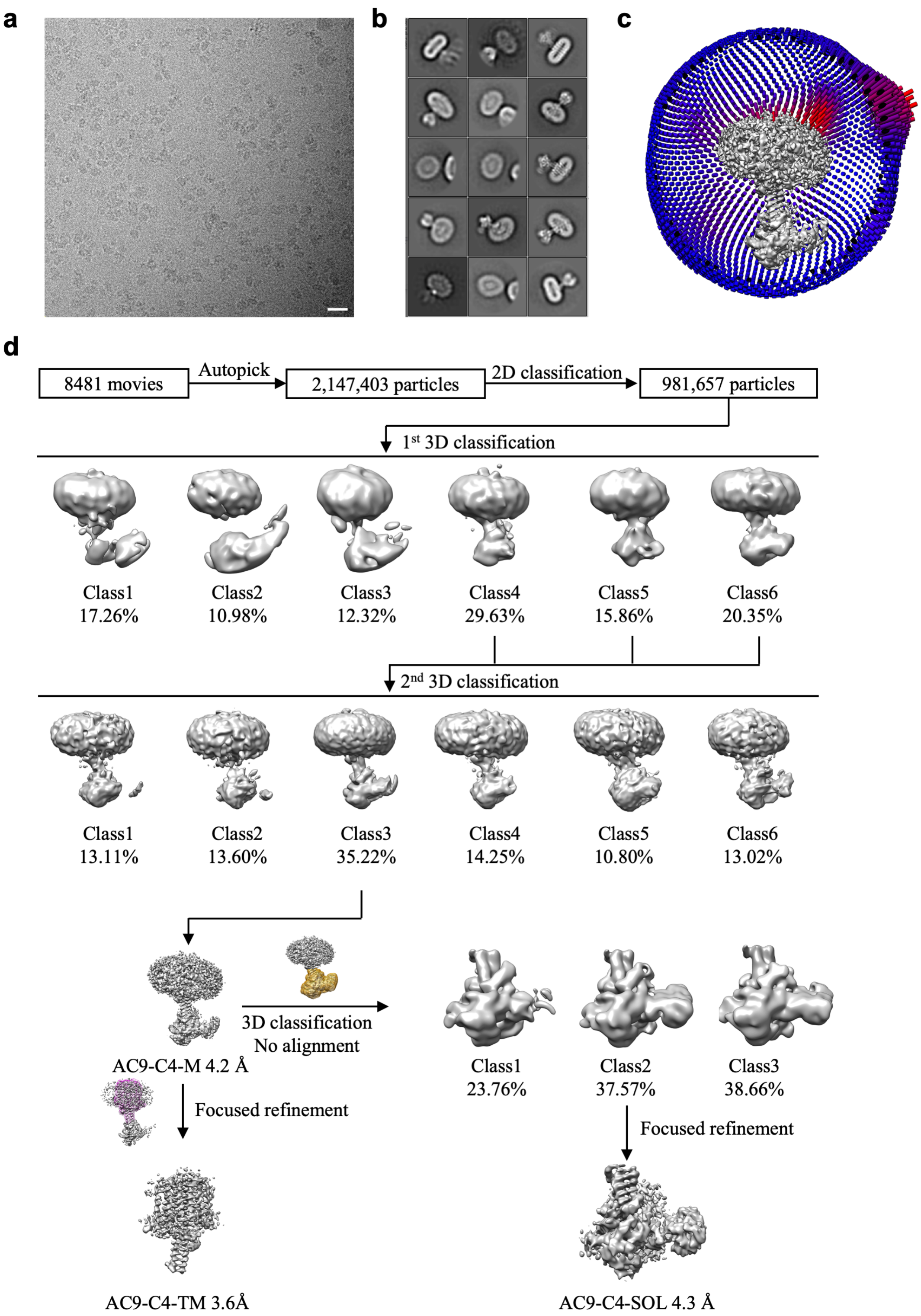

Supplementary Figure 6 | Cryo-EM image processing procedure for the AC9-C4-M dataset. a, A representative micrograph from 8481 micrographs of AC9-C4 complex in the presence of 0.5 mM MANT-GTP; scale bar corresponds to 20 nm. **b**, 2D classes of AC9-C4 dataset feature a relatively small density adjacent to the catalytic domain of AC9. **c**, Angular distribution of AC9-C4 dataset. **d**, Overview of the AC9-C4 dataset image processing procedure, including particle picking, 2D and 3D classification, 3D auto-refinement and focused refinement using the mask covering the TM region and the soluble region.

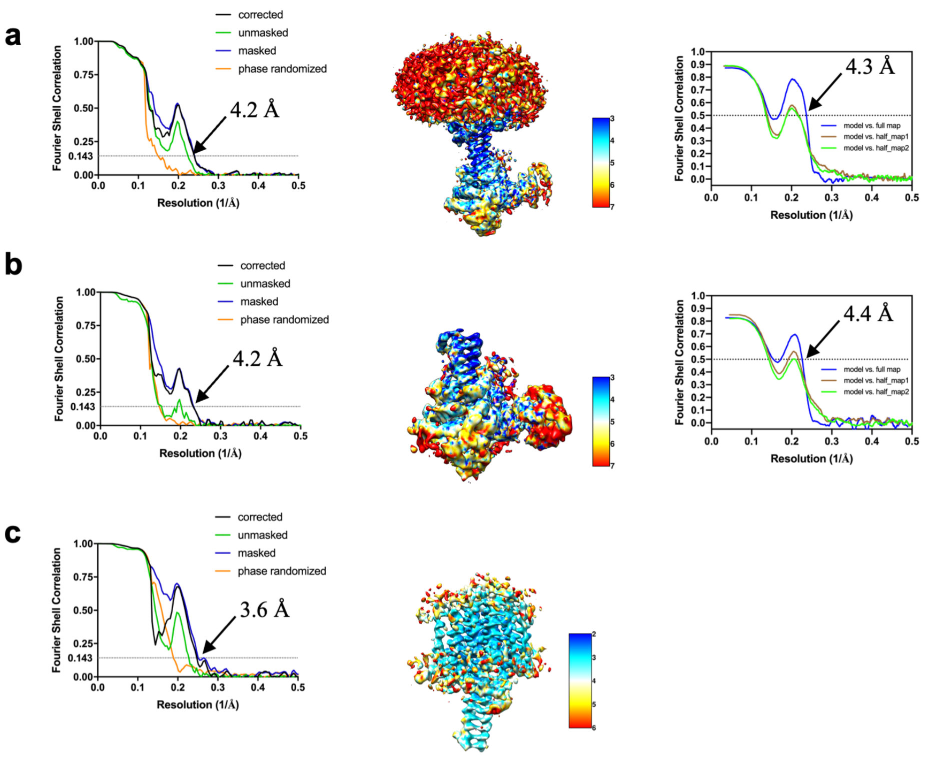

Supplementary Figure 7 | FSC plots and local resolution estimation for the 3D reconstruction of AC9-C4-M complex. a, AC9-C4-M complex. **b**, Focus refinement the soluble part of AC9-C4-M complex. **c,** Focus refinement the membrane part of AC9-C4-M complex. FSC plots are shown in the left panels, local resolution surface representations for each indicated map estimation are shown in the middle panels, and the map to model FSC plots are indicated in the right panels.

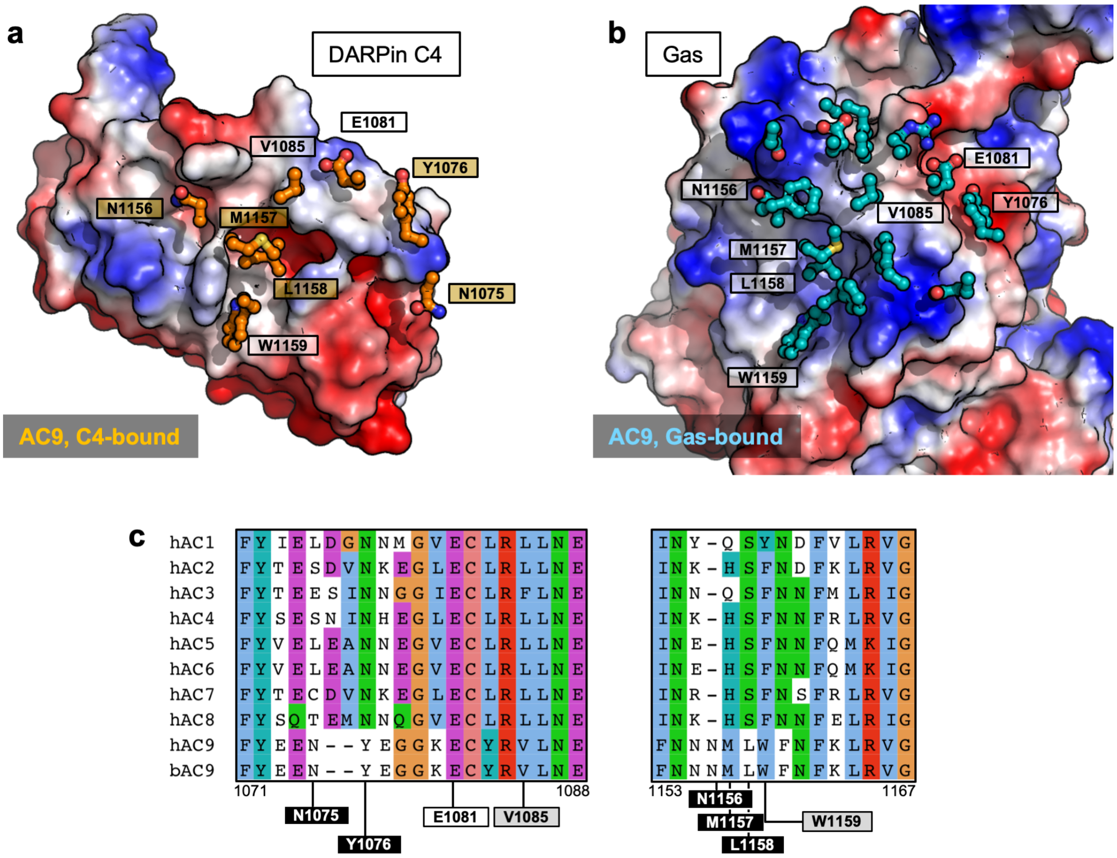

Supplementary Figure 8 | Unique residues of AC9 at the DARPin C4-binding interface. a, A view of the DARPin C4, in electrostatic potential-coloured surface representation. The residues of AC9 within 4 Å of the binder are shown as sticks. **b**, Same as in A, with Gαs protein in the AC9-Gαs complex shown in surface representation. **c**, Sequence alignment of the G protein-binding regions in human AC1-AC9 and bovine AC9. The residues unique for AC9 at the DARPin C4-AC9 interface are shown as block boxes (corresponding to the yellow boxes in a). The conserved substitutions are shown as grey boxes.

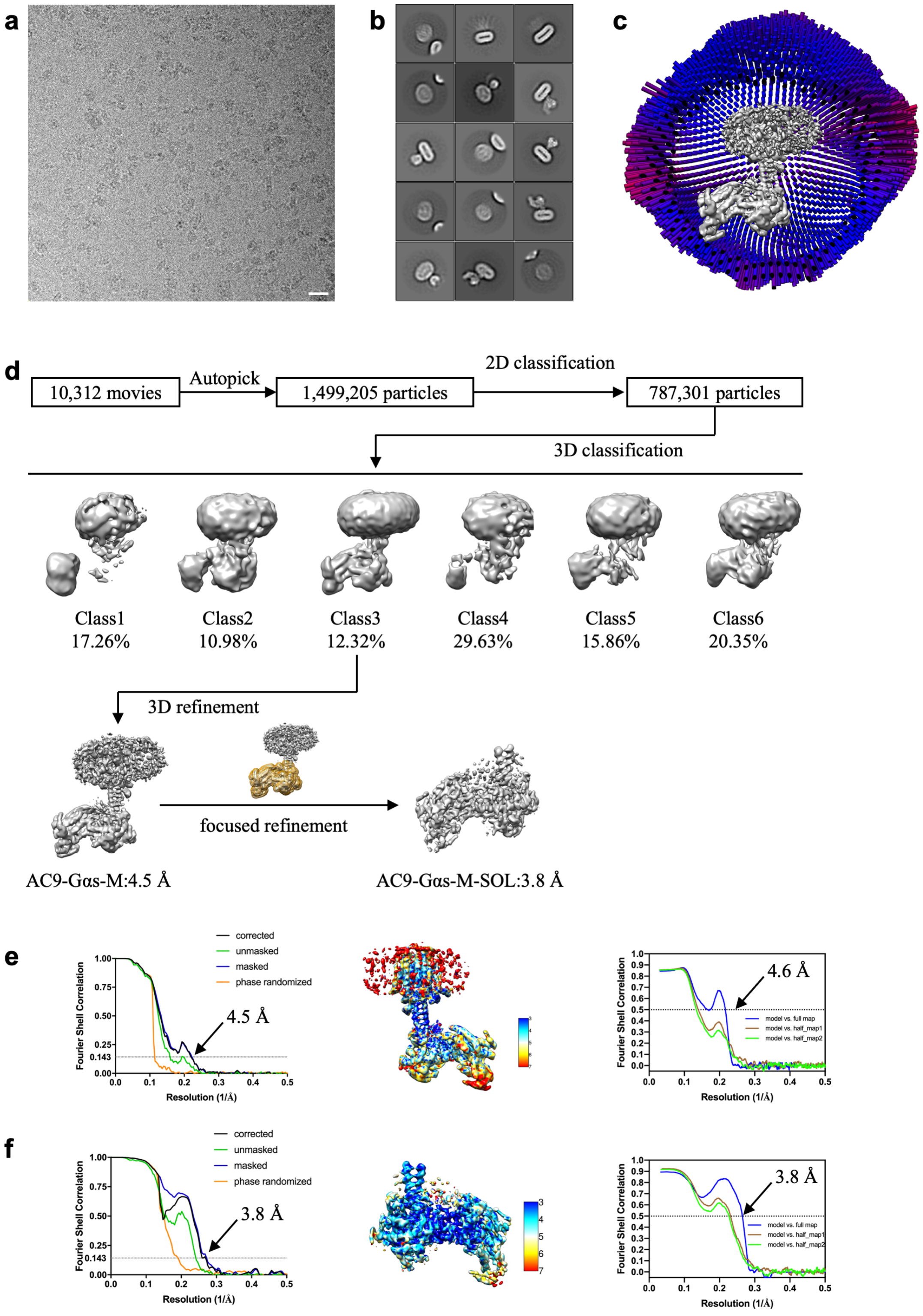

Supplementary Figure 9 | Cryo-EM image processing procedure of AC9$_{1250}$-Gαs-M dataset. a, A representative micrograph from 10312 micrographs of AC9$_{1250}$-Gαs complex in presence of 0.5 mM MANT-GTP; scale bar corresponds to 20 nm. **b**, 2D classes of AC9$_{1250}$-Gαs-M dataset. **c**, Angular distribution of AC9$_{1250}$-Gαs-M dataset. **d**, Overview of the image processing procedure, including particle picking, 2D and 3D classification, 3D auto-refinement and focus refinement using the mask

covering soluble region of the complex. **e-f**, FSC plots of map (left), local resolution estimation (*middle*) and map to model FSC plots (*right*) of $AC9_{1250}$-Gαs-M dataset.

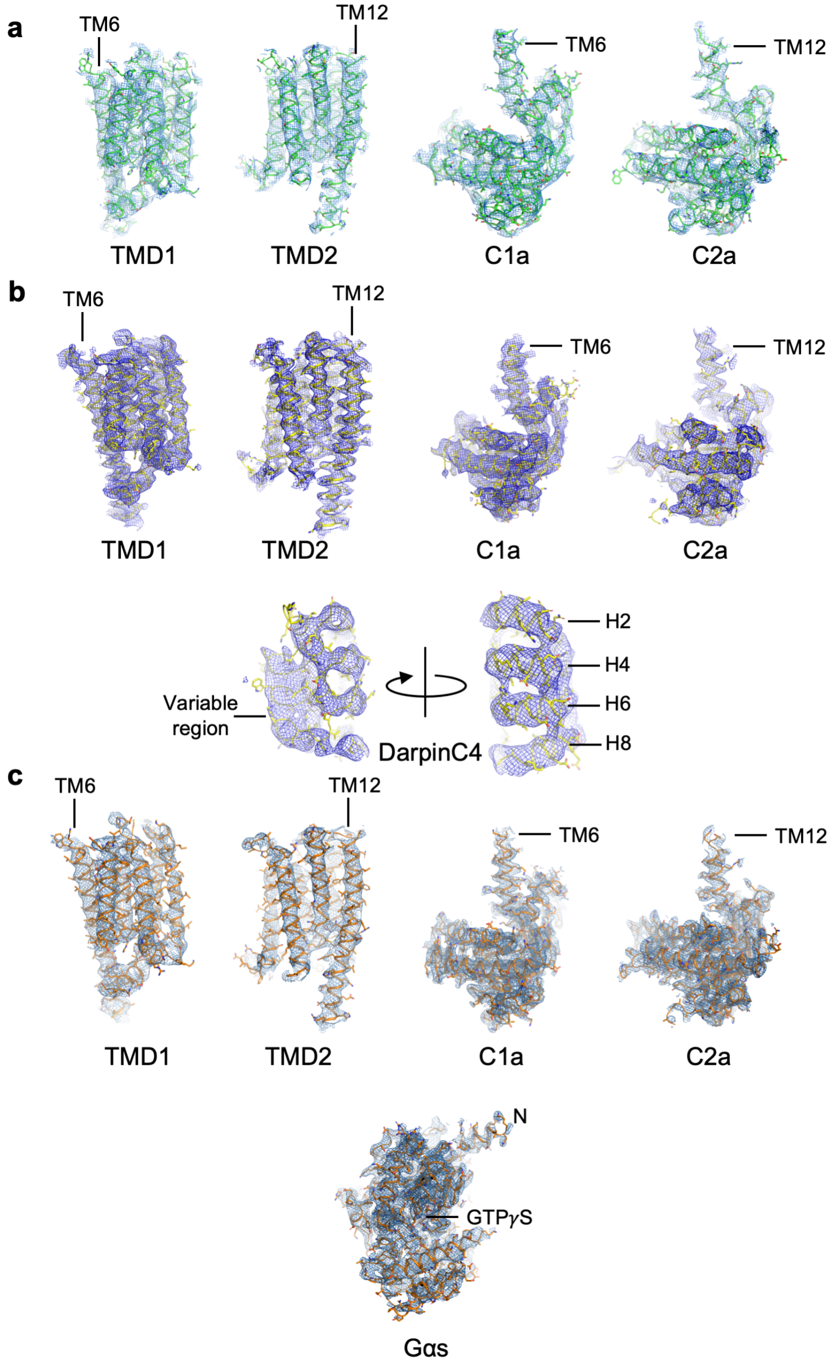

Supplementary Figure 10 | Features of the cryo-EM density maps. The density maps in mesh representation and the corresponding models (backbone represented as ribbons and side-chains represented as lines) are shown for: AC9-M (a), AC9-C4-M (b) and AC9-Gαs-M (c). The transmembrane (TMD1, TMD2) and catalytic domains (C1a, C2a), DARPin C4, Gαs subunit are shown seperately. Positions oft he TM6 and TM12 are indicated in the figure.

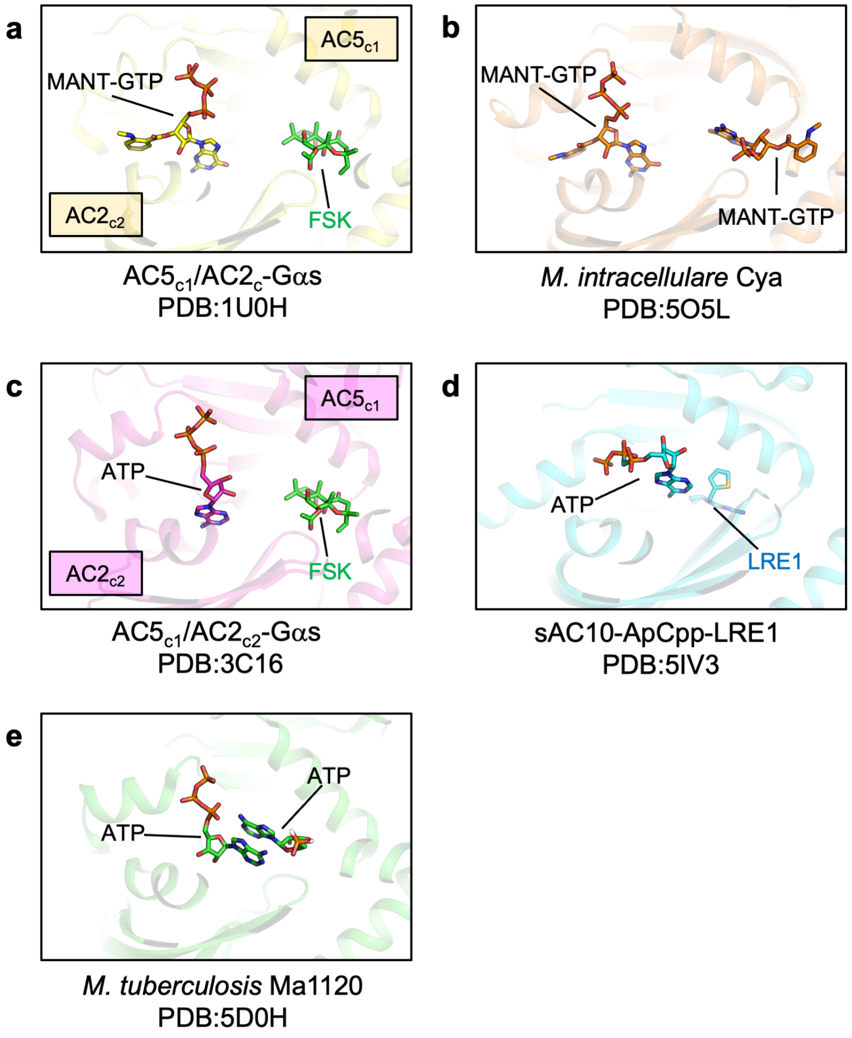

Supplementary Figure 11 | Comparison of nucleotide-bound states of AC9 with those of other ACs. a, The crystal structure of $AC5_{c1}/AC2_{c2}$-Gαs (PDB:1U0H) shows MANT-GTP is in M1 pose. **b**, The crystal structure of the *M. intracellulare* Cya (PDB:5O5L) shows MANT-GTP is in M1 pose. **c**, The structure of $AC5_{c1}/AC2_{c2}$-Gαs (PDB:3C16) shows ATP is in catalysis-compatible pose A2. **d**, The crystal structure of sAC-ApCpp-LRE1 (PDB: 5IV3) shows a non-canonical nucleotide pose in which adenine group interacts with the inhibitor LRE1. **e**, The structure of *M. tuberculosis* Ma1120 (PDB: 5D0H) features a non-canonical ATP pose, with adenine moieties of the two adjacent ATP molecules packing against each other.

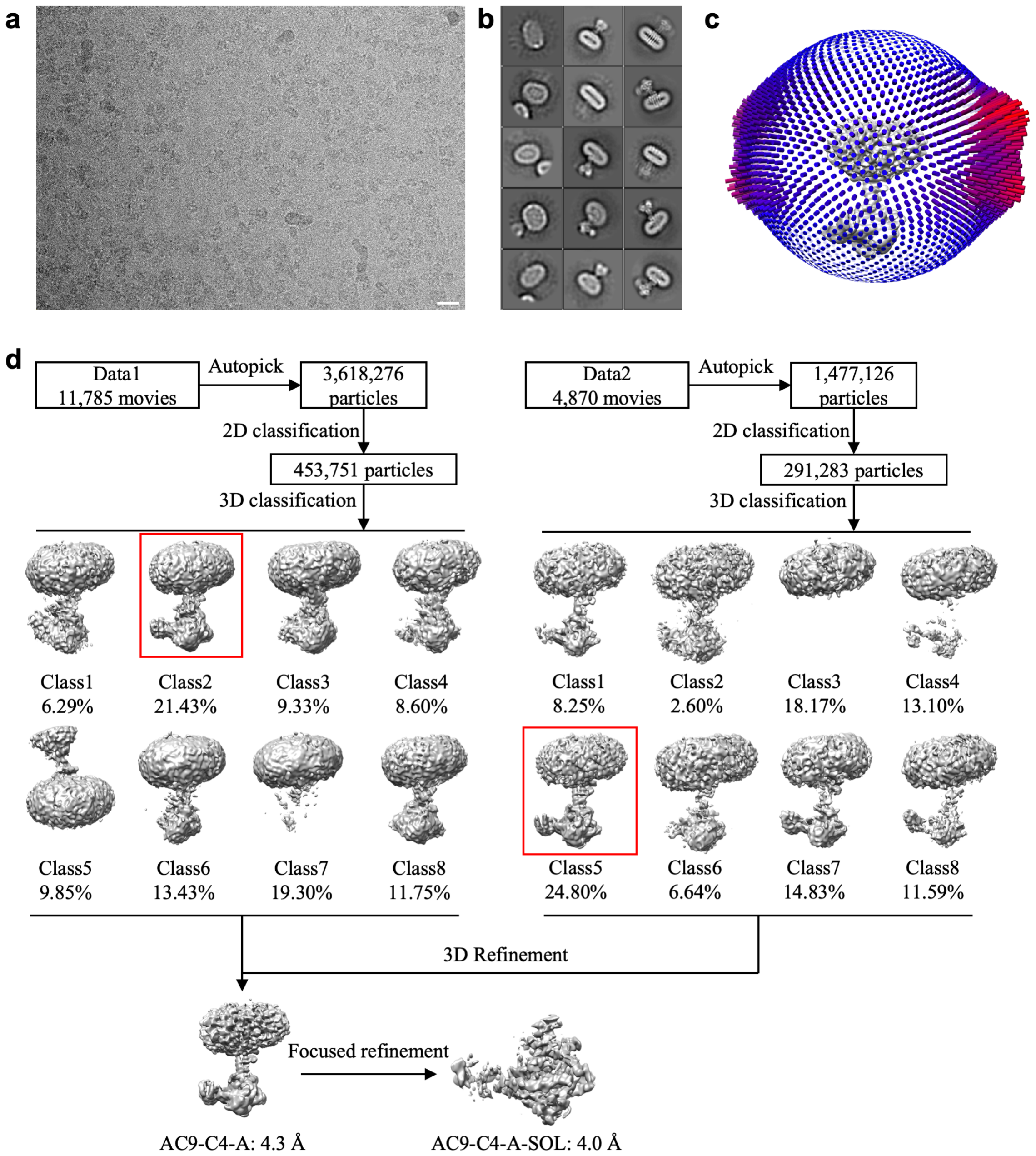

Supplementary Figure 12 | Cryo-EM image processing procedure for the AC9-C4-A dataset. a, A representative micrograph from 11785 micrographs of AC9-C4-A complex in the presence of 0.5 mM ATPαS; scale bar corresponds to 20 nm. **b,** 2D classes of AC9-C4-A dataset. **c,** Angular distribution of AC9-C4-A dataset. d, Overview of the AC9-C4-A dataset image processing procedure, including particle picking, 2D and 3D classification, 3D auto-refinement and focused refinement using the mask covering the soluble region.

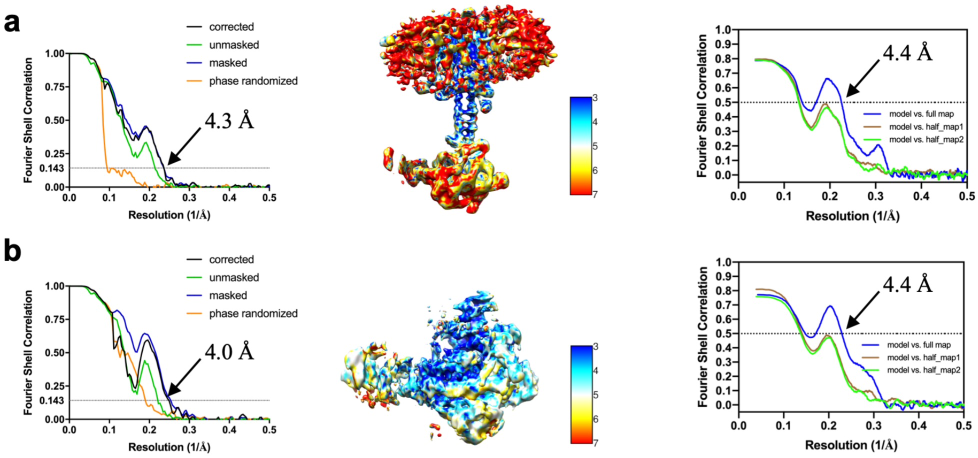

Supplementary Figure 13 | FSC plots and local resolution estimation for the 3D reconstruction of AC9-C4-A complex. FSC plots are shown in the left panels, local resolution surface representations for each indicated map estimation are shown in the middle panels, and the map to model FSC plots are indicated in the right panels. **a,** FSC plots and local resolution map of AC9-C4-A map. **b,** FSC plots and local resolution map of the focus refinement of AC9-C4-A-SOL map.

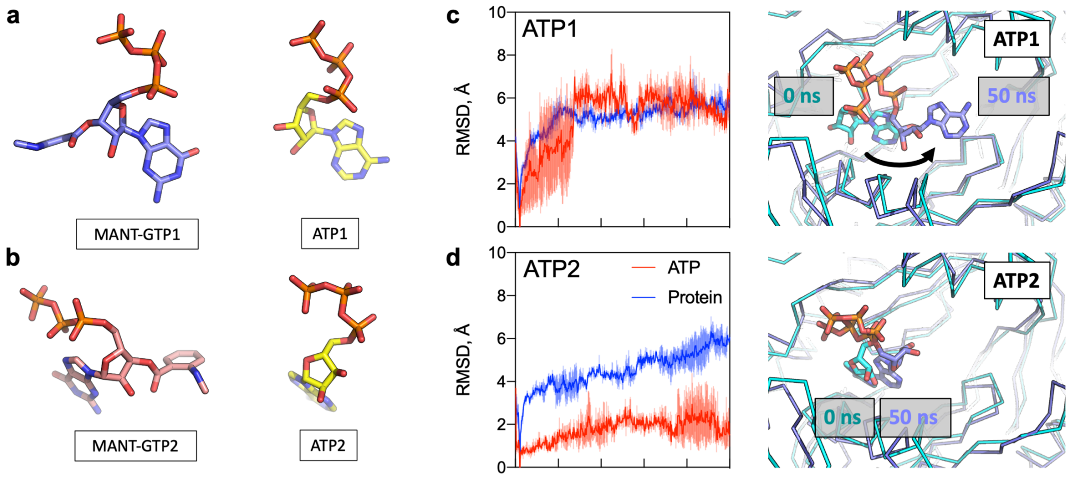

Supplementary Fig. 14 | Molecular dynamics simulation of the two possible ATP-bound states of AC9. a-b, The experimentally determined positions of the MANT-GTP molecules correspond to the theoretical substrate-bound (ATP-bound) states of AC9. **c-d**, The models of the AC9 catalytic domains bound to the molecules of ATP in two possible conformations (ATP1 and ATP2), modelled according to the experimentally determined structures of AC9 complexes, were subjected to MD simulations. The traces correspond to the atomic displacements of the protein (blue) or ATP molecules (red). The panels on the right indicate the starting (teal) and the end-states (blue) of each system after 50 ns equilibration. Although state ATP2 (d) shows a relatively stable conformation of ATP, the state ATP1 changes substantially, resulting in long-range displacement of ATP away from the active site (black arrow in c).

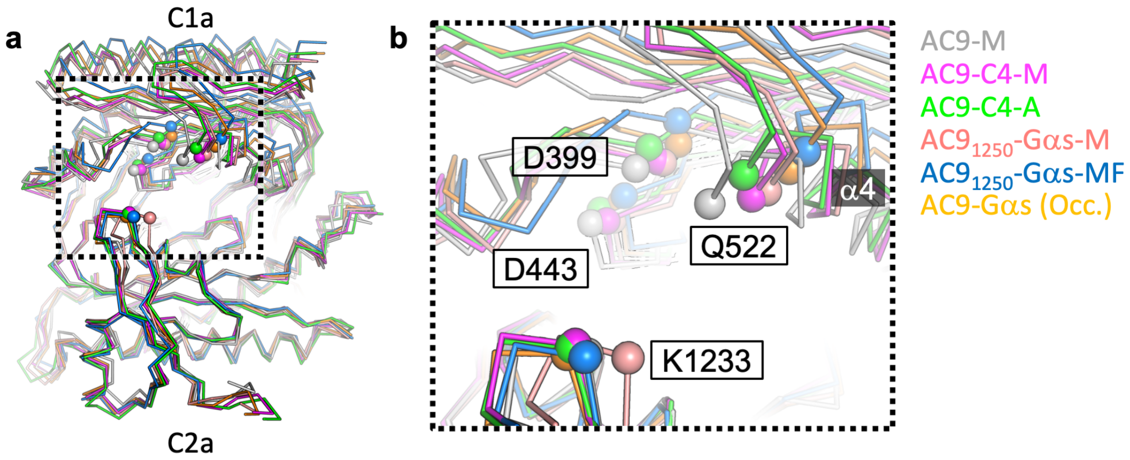

Supplementary Fig. 15 | Structural transitions in the distinct activator-bound states of AC9 in the presence of MANT-GTP and ATPαS. **a**, All available structures of AC9 were structurally aligned using the C2a domain as an anchor (as in Fig. 4e, with an additional structure, AC9-C4-A, coloured green). **b**, Comparison of the five available structures reveals relative displacement of the active site residues, D399, D443, K1233 and the residue Q522 in the helix α4 (Cα atoms are shown as spheres). The individual structures are indicated on the right side of the panel. Comparison of the MANT-GTP- (AC9-C4-M, magenta) and ATPαS-bound state (AC9-C4-A, green) shows a high degree of similarity between them. The distance between the Cα atoms of D399, D443 and Q522 residues in the C1a domain of these two structures is 1.1 Å, 1.4 Å and 1.8 Å, respectively.

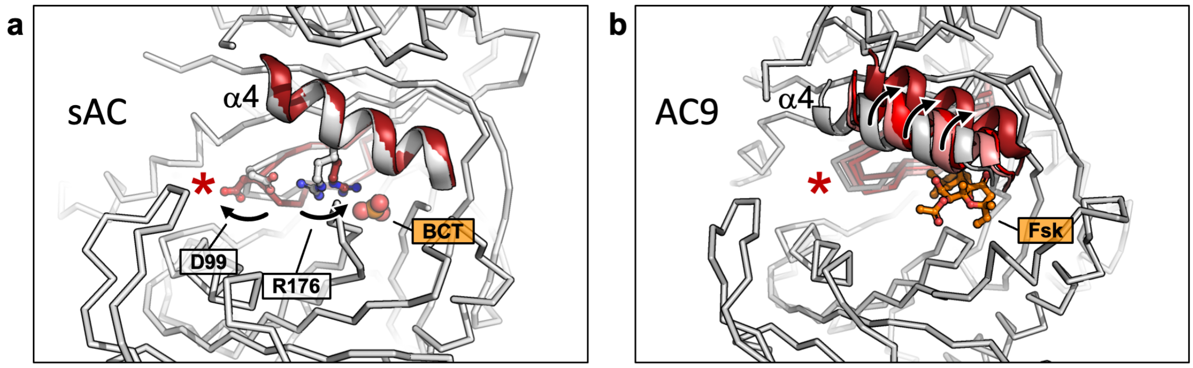

Supplementary Fig. 16 | Comparison of the activator-induced conformational changes in AC9 and in soluble AC, sAC. a. In the case of sAC, the helix α4 undergoes minimal changes upon binding of the bicarbonate ion (red α4 helix - bicarbonate-bound state PDB ID: 4cll; white - apo-state PDB ID: 4clf; the protein model in ribbon representation corresponds to the apo state). Instead of going through large-scale domain movements, a side chain of the residue R176 is drawn away from the side chain of D99, a residue that participates in coordinating the metal ions critical for catalysis. This switch mechanism underlies sAC activation by $HCO3^-$. **b**, The conformational changes in AC9 involve whole-domain rearrangements, with helix α4 (which participates in forskolin binding) moving concomitantly with the potency of the activating agent. The ribbon model corresponds to the AC9-M state. The progressively darker red α4 helices correspond to the states AC9-C4-M, AC9-Gas-M and AC9-Gαs-MF. The asterisk in a and b indicates protein activation.

Supplementary Table 1 | Cryo-EM data collection and refinement statistics.

**Data collection**							
Sample	AC9-M	AC9-C4-M		AC9$_{1250}$-Gαs-M		AC9-C4-A	
Instrument	FEI Titan Krios/Gatan K2 Summit/Quantum GIF					Gatan K3	
Voltage	300					300	
Electron dose(e^-/Å)	40					48	
Defocus range (μm)	-0.75 to -2.5					-1.0 to -2.0	
Pixel size (Å)	0.81					0.678	
Map resolution (Å) FSC threshold 0.143	AC9-M	AC9-C4-M-FL	AC9-C4-SOL	AC9-Gαs-M-FL	AC9-Gαs-M-SOL	AC9-C4-A-FL	AC9-C4-A-SOL
	4.9	4.2	4.2	4.5	3.8	4.3	4.0
Number of particles	141,446	210,729	210,729	157,200	157,200	170,731`	170,731
Refinement							
Model resolution (Å) FSC threshold 0.5	7.7	4.3	4.4	4.6	3.8	4.4	4.4
Map sharpening b-factor (Å)	-192.2	-204.7	-189.0	-197.83	-183.8	-100	-164.169
Map CC	0.73	0.78	0.75	0.8	0.82	0.66	0.69
Model composition							
Protein residues/ligand	842	970	577	1187/1	794/1	970	577
ADP (B factor)	351.53	149.98	138.11	205.14	76.56	199.29	164.22
Bond length r.m.s.d. (Å)	0.005	0.009	0.004	0.006	0.008	0.005	0.002
Bond length r.m.s.d. (°)	0.866	1.075	0.741	0.779	0.818	0.819	0.644
Validation							
MolProbity score	2.66	2.62	2.26	2.48	2.27	2.07	1.93
Clash score	38.14	33.32	20.58	29	16.33	16.04	11.3
Rotamer outliers (%)	0	1.09	0	0.19	0.29	0	0
Ramachandran plot							
Favored (%)	88.29	88.78	92.95	90.76	89.9	94.76	94.71
Allowed (%)	11.71	11.22	7.05	9.24	10.1	5.24	5.29
Disallowed (%)	0	0	0	0	0	0	0

Supplementary References

1. C. Qi, S. Sorrentino, O. Medalia, V. M. Korkhov, The structure of a membrane adenylyl cyclase bound to an activated stimulatory G protein. *Science* **364**, 389-394 (2019).
