# Supplementary material for: Structural basis of adenylyl cyclase 9 activation
Source: Nat Commun. 2022 Feb 24;13:1045. doi: 10.1038/s41467-022-28685-y (PMC8873477; doi:10.1038/s41467-022-28685-y)
Supplement: Supplementary file 3 — Description of Additional Supplementary Files [file 41467_2022_28685_MOESM3_ESM.pdf]

Description of Additional Supplementary Files

File name: Supplementary Movie 1

Description: Molecular dynamics simulation of ATP1 state. ATP1 shifts from its original location.

File name: Supplementary Movie 2

Description: Molecular dynamics simulation of ATP2 state. ATP2 shows a relatively stable conformation in 50 ns of the simulation.

File name: Supplementary Movie 3

Description: Molecular morph of AC9 in different states. AC9-C4 (magenta, partial activated state), AC9-Gαs (orange, occluded state), $AC9_{1250}$- Gαs-M (pink, partial activated state) and $AC9_{1250}$-Gαs-MF (blue, fully activated state) are aligned to AC9- M (white, basal state) based on C2a domain. The whole-domain rearrangements correspond to increased levels of AC9 activation.
