# Supplementary material for: Structural basis of adenylyl cyclase 9 activation
Source: Nat Commun. 2022 Feb 24;13:1045. doi: 10.1038/s41467-022-28685-y (PMC8873477; doi:10.1038/s41467-022-28685-y)
Supplement: Supplementary file 7 — Reporting Summary [file 41467_2022_28685_MOESM7_ESM.pdf]

nature research

Corresponding author(s): Volodymyr Korkhov

Last updated by author(s): Jan 8, 2022

Reporting Summary

Nature Research wishes to improve the reproducibility of the work that we publish. This form provides structure for consistency and transparency in reporting. For further information on Nature Research policies, see our Editorial Policies and the Editorial Policy Checklist.

Statistics

For all statistical analyses, confirm that the following items are present in the figure legend, table legend, main text, or Methods section.

n/a	Confirmed	
☐	☒	The exact sample size (*n*) for each experimental group/condition, given as a discrete number and unit of measurement
☒	☐	A statement on whether measurements were taken from distinct samples or whether the same sample was measured repeatedly
☐	☒	The statistical test(s) used AND whether they are one- or two-sided<br>*Only common tests should be described solely by name; describe more complex techniques in the Methods section.*
☒	☐	A description of all covariates tested
☒	☐	A description of any assumptions or corrections, such as tests of normality and adjustment for multiple comparisons
☐	☒	A full description of the statistical parameters including central tendency (e.g. means) or other basic estimates (e.g. regression coefficient) AND variation (e.g. standard deviation) or associated estimates of uncertainty (e.g. confidence intervals)
☐	☒	For null hypothesis testing, the test statistic (e.g. *F*, *t*, *r*) with confidence intervals, effect sizes, degrees of freedom and *P* value noted<br>*Give P values as exact values whenever suitable.*
☒	☐	For Bayesian analysis, information on the choice of priors and Markov chain Monte Carlo settings
☒	☐	For hierarchical and complex designs, identification of the appropriate level for tests and full reporting of outcomes
☒	☐	Estimates of effect sizes (e.g. Cohen's *d*, Pearson's *r*), indicating how they were calculated

Our web collection on statistics for biologists contains articles on many of the points above.

Software and code

Policy information about availability of computer code

Data collection	EPU2.0, SerialEM3.6
Data analysis	GraphPad Prism 8, Relion-3.0, Motioncor2, Gctf1.06, phenix1.17, pymol2.1, chimera 1.15, coot 0.8.9.2, Gromacs-2019.2, VMD1.9.4a38, ImageJ2.3.0, ResMap1.1.4.

For manuscripts utilizing custom algorithms or software that are central to the research but not yet described in published literature, software must be made available to editors and reviewers. We strongly encourage code deposition in a community repository (e.g. GitHub). See the Nature Research guidelines for submitting code & software for further information.

Data

Policy information about availability of data

All manuscripts must include a data availability statement. This statement should provide the following information, where applicable:

- Accession codes, unique identifiers, or web links for publicly available datasets
- A list of figures that have associated raw data
- A description of any restrictions on data availability

The cryo-EM density maps have been deposited in the Electron Microscopy Data Bank, with accession numbers EMD-13330, EMD-13331, EMD-13334, EMD-13335, EMD-13336, EMD-13337 and EMD-13338. The coordinates have been deposited in the Protein Data Bank, with entry codes PDB:7PD4, PDB:7PD8, PDB:7PDD, PDB:7PDE, PDB:7PDF, PDB:7PDG and 7PDH. All materials are available upon request. Source data are provided with this paper.

Field-specific reporting

Please select the one below that is the best fit for your research. If you are not sure, read the appropriate sections before making your selection.

☒ Life sciences ☐ Behavioural & social sciences ☐ Ecological, evolutionary & environmental sciences

For a reference copy of the document with all sections, see nature.com/documents/nr-reporting-summary-flat.pdf

Life sciences study design

All studies must disclose on these points even when the disclosure is negative.

Sample size	Adenylyl cyclase activity assays were performed at least three times (or more) to ascertain the reproducibility of the experiments. No statistical methods were used to predetermine the sample size. The sample size was determined based on reproducibility and sufficiency for accurate measurements.
Data exclusions	Standard single particle analysis (2D and 3D classification) was used to exclude /select the particles for subsequent 3D reconstruction and refinement.
Replication	Where appropriate three or more experiments were performed to ascertain the reproducibility of the experiments. Replication of the results was successful.
Randomization	Allocation of the samples in groups was random.
Blinding	The knowledge of dataset or group assignment was not judged to influence the outcome of data analysis (and to not introduce a bias in the interpretations of the results). Therefore, blinding was not performed.

Reporting for specific materials, systems and methods

We require information from authors about some types of materials, experimental systems and methods used in many studies. Here, indicate whether each material, system or method listed is relevant to your study. If you are not sure if a list item applies to your research, read the appropriate section before selecting a response.

Materials & experimental systems

n/a	Involved in the study
☐	☒ Antibodies
☐	☒ Eukaryotic cell lines
☒	☐ Palaeontology and archaeology
☒	☐ Animals and other organisms
☒	☐ Human research participants
☒	☐ Clinical data
☒	☐ Dual use research of concern

Methods

n/a	Involved in the study
☒	☐ ChIP-seq
☒	☐ Flow cytometry
☒	☐ MRI-based neuroimaging

Antibodies

Antibodies used	MAb Anti FLAG M2-d2, Cisbio, Cat. number selected : 5000 Tests (61FG2DLA)
Validation	MAb Anti FLAG M2-d2 is an IgG1 raised against FLAG® fusion proteins labeled with d2. Unlike anti-FLAG®M1 antibody, the M2 antibody will recognize the FLAG® sequence at the N-terminus or C-terminus of FLAG® fusion proteins.<br>This reagent can be used in both biochemical and cellular formats to study a wide variety of interactions: protein/protein, protein/peptide, protein/DNA, protein/RNA, protein/carbohydrate, protein/small molecule, receptor/ligand<br>HTRF can detect a broad range of affinity constants ranging from picomolar to low millimolar (https://ch.cisbio.eu/mab-anti-flag-m2-d2-40028)

Eukaryotic cell lines

Policy information about cell lines

Cell line source(s)	HEK293F cell line were obtained from ThermoFisher,Catalog number: R79007. High five cells were obtained from ThermoFisher, Catalog number: B85502. Sf9 cells were obtained from ThermoFisher, Catalog number: A35243.
Authentication	No further authentication was performed for commercial cell lines.

Mycoplasma contamination	HEK293F cells were tested for mycoplasma using PCR-tests (negative). High five cells and sf9 cells were not tested for mycoplasma.
Commonly misidentified lines (See ICLAC register)	No commonly misidentified lines.
